# Perceived healthiness of foods, food avoidance and diet-related anxiety in individuals with self-reported irritable bowel syndrome: a cross-sectional study

**DOI:** 10.1186/s40795-024-00945-8

**Published:** 2024-10-10

**Authors:** Sanna Nybacka, Anton Kinnander, Hanna Augustin, Linnea Bärebring

**Affiliations:** 1 Department of Molecular and Clinical Medicine, Sahlgrenska Academy, University of Gothenburg, Box 459, Gothenburg, SE-405 30 Sweden; 2 Scandinavian Gastro Clinic, Gothenburg, Sweden; 3 Department of Internal Medicine and Clinical Nutrition, Institute of Medicine, Sahlgrenska Academy, University of Gothenburg, Gothenburg, Sweden

**Keywords:** Irritable bowel syndrome, Food avoidance, Healthiness, Anxiety

## Abstract

**Background:**

Irritable bowel syndrome (IBS) is a common gastrointestinal disorder in which the intake of food is known to exacerbate symptoms. Experiencing food related symptoms can lead to avoidance of food, and cause anxiety related to food intake. We aimed to explore perceptions of the healthiness of food, food avoidance, and food-related worry and anxiety among individuals with and without IBS.

**Methods:**

This study was based on a survey conducted in January-February 2017. In total, 2000 participants aged 20–65 were invited by postal letter randomly obtained through the Swedish state personal address register. The questionnaire included aspects of socioeconomic position, different disorders including IBS and food intolerances, food avoidance, and food-related worry or anxiety.

**Results:**

In total, 538 participants were included in this study, of whom 8.4% (*n* = 45) reported having IBS. There were few differences regarding the perceived healthiness of foods between individuals with and without IBS. Participants with IBS avoided gluten (OR 3.45, *p* = 0.002), lactose (OR 5.0, *p* < 0.001) and alcohol (OR 2.0, *p* = 0.042) more frequently than individuals without IBS, and avoidance was driven by abdominal pain. Participants with IBS also reported feeling more worried and having anxiety about diet than those without IBS (*p* = 0.032 and *p* = 0.002, respectively).

**Conclusion:**

This study indicates that individuals with IBS perceive most foods as equally healthy as individuals without IBS. Having IBS increases the odds of avoiding gluten, lactose, and alcohol. Worry and anxiety related to diet were more common among individuals with IBS, and these aspects need to be considered both in clinical practice and in future research.

**Supplementary Information:**

The online version contains supplementary material available at 10.1186/s40795-024-00945-8.

## Introduction

Irritable bowel syndrome (IBS) is a disorder of gut-brain interactions, characterized by abdominal pain, bloating, and altered bowel habits [1]. It is one of the most common gastrointestinal disorders, with a global prevalence ranging from 3.8 to 9.2%, depending on local variations and the diagnostic criteria used [2, 3]. The diagnosis of IBS is currently based on the Rome IV criteria, which require the absence of abnormal organic or biochemical features that can explain the symptoms [4]. IBS is more prevalent in women than men, and the prevalence decreases with age [2]. Although the pathophysiology of IBS is not fully understood, several key factors have been identified, including gastrointestinal dysmotility [5], visceral hypersensitivity [5] and dysfunctions in gut-brain interactions [6]. The gut-brain axis is believed to have great significance, as the communication between the gut and brain is reciprocal, and disruptions in their interaction can have numerous cascading effects [7]. Emotional states, such as anxiety, may slow down or stimulate gastrointestinal motility [8], which in turn can lead to urgency and/or diarrhea [9]. Psychological stress is another important factor, as it affects visceral sensitivity and intestinal motility [6, 9]. The prevalence of anxiety disorders in patients with IBS is over 30%, and patients with IBS have three times higher odds of having an anxiety disorder than healthy individuals [10, 11].

Dietary treatment is included in the first-line treatment option for IBS, and currently based on the NICE guidelines [12]. In short, the guidelines focus on healthy dietary patterns and a reduction in known triggering dietary factors such as caffeine, carbonated beverages, alcohol, high fiber foods, and resistant starch. If first-line dietary modifications fail to reduce IBS symptoms, a more restrictive dietary approach may be utilized, such as a diet that is low in fermentable oligo-, di-, and mono-saccharides, and polyols (FODMAPs) [13]. FODMAPs are short-chained carbohydrates that are poorly digested and thus are fermented by bacteria in the colon [13]. Some FODMAPs are also osmotically active, which leads to an increase in intraluminal water volume and distention [14]. The low FODMAP diet has become more widely used as a treatment option with increasing evidence of its efficacy [15, 16]. However, restrictive diets have lately been criticized, as restricting the intake of certain foods increases the risk of nutritional deficiencies [17] and might lead to unwanted food avoidance behaviors [18]. Individuals who are at risk of disordered eating are thus advised against diets that are restrictive in their nature [19].

Food-related symptoms are often reported in IBS, whereby a common strategy of avoiding symptoms is to avoid intake of certain foods [18]. Perceptions of the healthiness or harmfulness of specific food items can significantly influence dietary patterns and contribute to the development of food avoidance strategies. A recent study showed that patients with IBS who reported food avoidance and restriction have reduced quality of life, worse overall symptoms, and more psychological distress [18]. However, it remains unclear whether food avoidance in IBS is solely driven by the fear of gastrointestinal symptoms or also by broader concerns related to health consciousness. Individuals with IBS may be particularly sensitive to how their food choices impact their symptoms, which could lead to altered eating habits and further affecting their quality of life. In this study, we therefore aimed to investigate the perceived healthiness of specific foods or food components and food avoidance in individuals with and without IBS. Second, we aimed to study differences in worry and anxiety related to health aspects of foods in individuals with and without IBS.

## Materials and methods

### Study design

This study utilized a cross-sectional survey conducted between January and February 2017 on beliefs and attitudes toward diet and health in Sweden – the SWEDIET-2017 survey. The survey has been described in detail in previous publications [20, 21]. Recruitment of participants was done by post using a random selection from the Swedish state personal address register that includes addresses of all persons who are registered as residents in Sweden. Participants from all parts of Sweden, between the ages of 20–65 years, were eligible. This age range was chosen to primarily recruit participants with autonomy over their dietary intake. The exclusion criteria were classified personal information or residents who did not have a registered Swedish address. Invitations were sent to 2,000 individuals on one occasion with no reminders. All participants were informed in writing about the study and that returning the completed questionnaire was regarded as giving informed consent to participate. Hence, all participants provided their written informed consent. All questionnaires were completely anonymous, and data could not be traced back to the individual participants. Thus, no reminder was posted. The survey was approved by the Regional Ethics Committee in Gothenburg, Sweden.

### Data collection

The questionnaire was six pages long and took approximately 10–15 min to complete. Completed questionnaires with more than 20% missing data or a nonbinary gender identity were excluded from the current analyses. The questionnaire included demographic variables such as gender, age, income, level of education, and occupation and general health questions including self-reported weight, height, and physical activity. The disorders or diseases queried in this survey included *IBS*,* gluten intolerance*,* lactose intolerance*,* other food allergies (open-ended)*,* obesity* and *rheumatoid arthritis*, and *an open-ended question for other diseases*, and participants could select all health conditions they identified as having. To control for the fact that the diagnosis of IBS was self-reported, the participants also answered the statement “*I often have stomach pain or discomfort*”, with answer options of *agree completely*,* partly agree*,* partly disagree*,* disagree completely or no opinion*. Nonrespondents on the IBS question or individuals who reported having IBS while having no stomach pain or discomfort were excluded.

Questions on perceived healthiness and avoidance of specific foods or food components were posed for carbohydrates, sugar, white flour, gluten, fat, dairy products, lactose, alcohol, red meat, food additives, and salt. Questions were formulated as *“What is your perception of the following dietary components?”* with answering options of *very unhealthy*,* partly unhealthy*,* partly healthy*,* very healthy or no opinion*. These food items were chosen primarily as they are commonly mentioned in dietary recommendations and in popular diets (such as clean eating or anti-inflammatory diets). Regarding food avoidance, the question was stated as *“Which of the following dietary components do you avoid because you perceive it to be unhealthy?”*. Participants were also asked to agree or disagree with the following statements regarding health and diet: *I worry that my diet is unhealthy*, and *I often have anxiety over my diet being unhealthy*. The options provided were *agree completely*,* partly agree*,* partly disagree*,* disagree completely or no opinion*.

Due to the small number of participants with IBS, the answer options *agree completely* and *partly agree* were merged into one category, *agree*, along with the categories *partly disagree* and *disagree completely*, which were merged into *disagree*.

Before the survey was conducted, the questionnaire was tested for clarity in a convenient sample of 10 participants in a wide age range which showed that the questions were easy to understand and perceived similarly among the respondents. Only few adjustments in language and age categories were needed.

### Statistical analysis

Frequencies and valid % of participants are presented for categorical data. To test for differences between participants with and without IBS, chi-square tests were used. When > 20% of cells were below the expected numbers, Fisher’s exact test was used. To analyze differences regarding avoidance of food components between participants with and without IBS, logistic regression was used with the food items as dichotomous variables, with 0 = *do not avoid* and 1 = *avoid*. To control for potential confounding factors, adjusted analyses were performed using multivariable logistic regression, adjusted for sex, BMI, and educational level, presented with odds ratios (OR) and confidence intervals (95% CI). Furthermore, sensitivity analyses were performed in which avoidance of lactose was conducted excluding individuals with self-reported lactose intolerance, and avoidance of gluten and white flour were analyzed excluding individuals who reported to be gluten intolerant. All statistical analyses were performed in IBS SPSS Statistics 26 (Armonk, New York: IBM Corp.), and significance was accepted at *p* < 0.05.

## Results

**Fig. 1:**
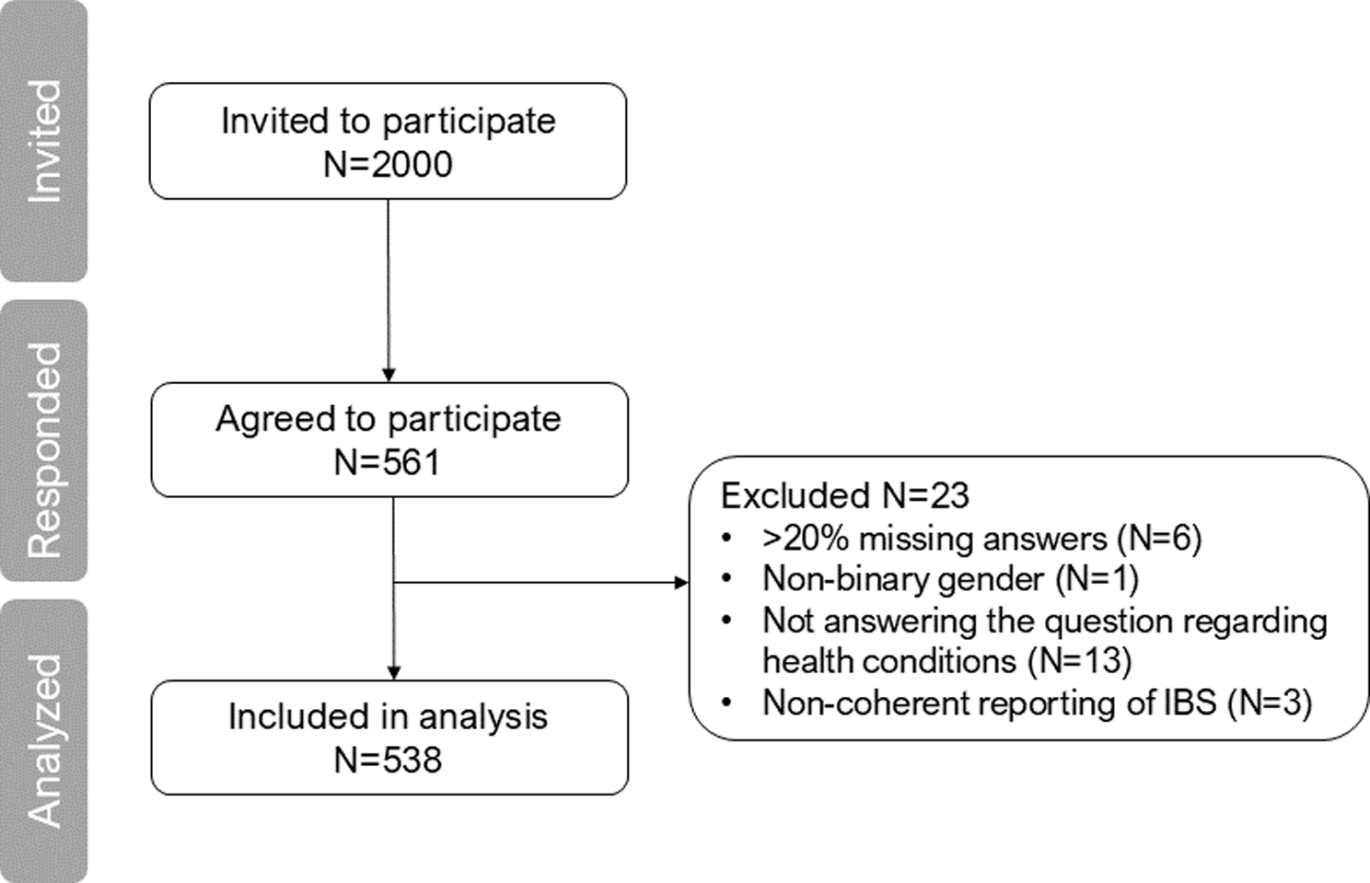
Flow chart of the study recruitment and data collection

A total of 561 completed questionnaires were returned, and the response rate was 28%. A total of 23 individuals were excluded; thus, 538 were included in this study (Fig. 1). The study population characteristics are presented in Table 1. Of all included participants, 56% were women, 19% reported having abdominal pain or discomfort without having IBS, and 8% reported having IBS. Approximately half of the participants had a university degree (53%), and a majority had full-time employment (67%). Among participants with IBS, 84% were women, which was proportionally larger compared to the non-IBS group (*p* < 0.001). Having abdominal pain or discomfort was significantly more prevalent among participants with IBS, as expected. Among participants with IBS, 49% completely agreed with having regular abdominal pain/discomfort, whereas 51% partially agreed. The corresponding numbers among the non-IBS participants were 3% and 33%, respectively. The prevalence of self-reported lactose intolerance, gluten intolerance, and food allergy was between 2.3 and 7 times higher among participants with IBS than among non-IBS participants.

**Table 1:** Characteristics of the study population divided into non-IBS individuals and individuals with IBS

	Non-IBS individuals *n* = 493	Individuals with IBS *n* = 45	*p* value^a^
**Women**,** n (%)**	261 (52.9)	38 (84.4)	< 0.001
**Age (years)**,** n (%)**			0.721
20–30	64 (13.0)	5 (11.1)	
31–45	137 (27.8)	15 (33.3)	
45–66	291 (59.0)	25 (55.6)	
**BMI**,** kg/m**^**2**^, **n (%)**			0.602
<25	257 (52.1)	25 (55.6)	
25–30	163 (33.1)	12 (26.7)	
>30	68 (13.8)	8 (17.8)	
**Income (SEK)**,** n (%)**			0.089
<10 000	38 (7.7)	6 (13.3)	
10 000–25 000	115 (23.3)	13 (28.9)	
25 000–40 000	226 (45.8)	22 (48.9)	
>40 000	111 (22.5)	4 (8.9)	
**Education**,** n (%)**			0.225
Primary	32 (6.5)	0	
Secondary	182 (36.9)	16 (35.6)	
University	258 (52.3)	27 (60.0)	
Other higher education	14 (2.8)	2 (4.4)	
**Employment**,** n (%)**			0.764
Fulltime	333 (67.4)	28 (62.2)	
Part-time	74 (15.0)	9 (20.0)	
Parental leave	8 (1.6)	1 (2.2)	
Student	20 (4.0)	1 (2.2)	
Other	58 (11.7)	6 (13.3)	
**Having frequent abdominal pain or discomfort**,** n (%)**			< 0.001
Agree	13 (2.6)	22 (48.9)	
Partially agree	164 (33.3)	23 (51.1)	
Disagree	310 (57.6)	NA	
No opinion	6 (1.1)	0	
**Reported lactose intolerance (yes)**,** n (%)**	37 (7.5)	10 (22.2)	0.003
**Reported gluten intolerance (yes)**,** n (%)**	9 (1.8)	6 (13.3)	< 0.001
**Reported food allergy (yes)**,** n (%)**	33 (6.7)	7 (15.6)	0.040

*Abbreviations* IBS, irritable bowel syndrome; BMI, body mass index

^a^ p value for IBS compared to non-IBS group

### Perceived healthiness of foods or food components

**Fig. 2:**
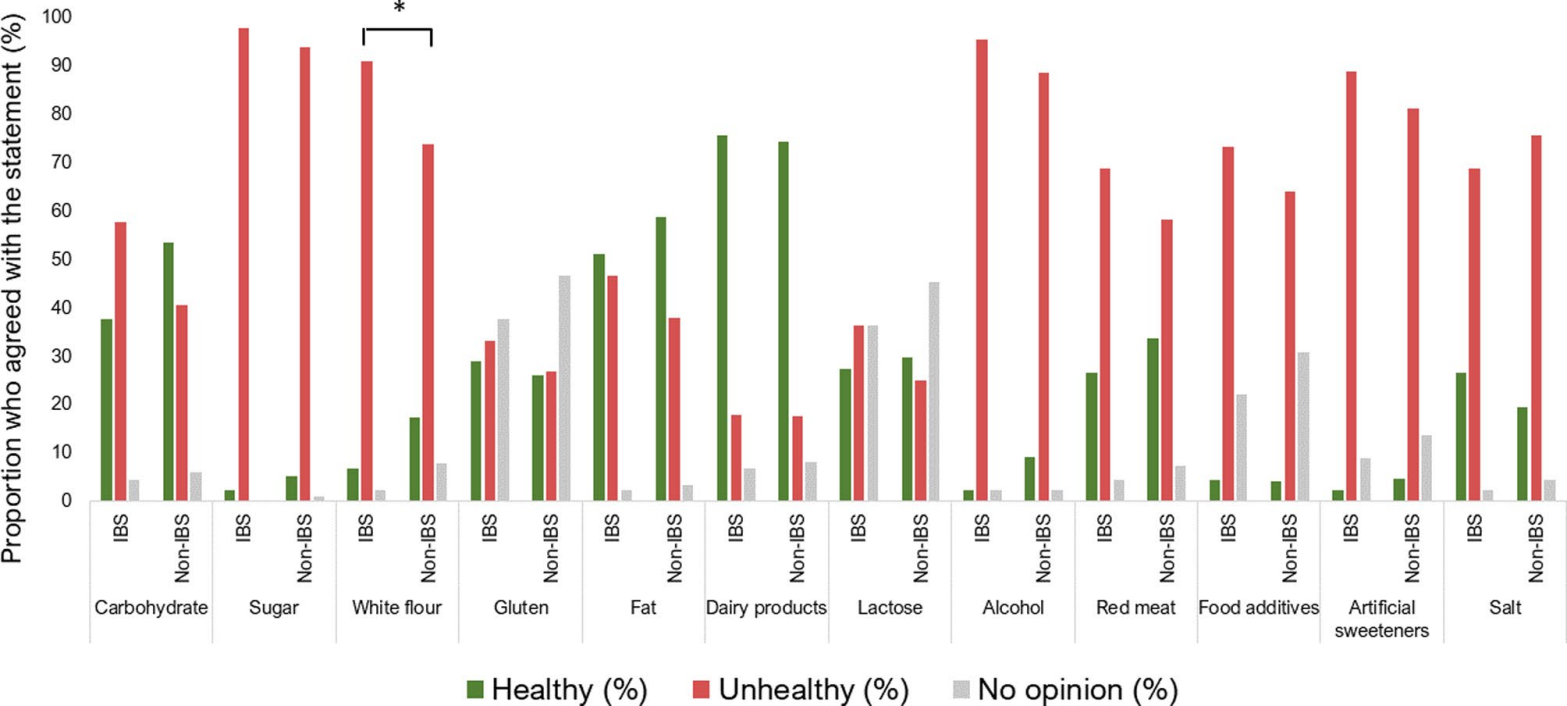
The proportion (%) of participants with irritable bowel syndrome (IBS) who perceived foods or food components as healthy or unhealthy was similar to that of individuals without IBS, except for white flour. Participants with IBS *N* = 45, and participants without IBS, *N* = 493. *p values < 0.05

Regarding the perceived healthiness of foods or food components among all study participants, dairy products were the food group that many considered healthy (74%), followed by fats (58%) and carbohydrates (52%). A majority of participants perceived sugar (94%) and alcohol (89%) as unhealthy. White flour was the only food group that differed in regards to perceived healthiness between participants with and without IBS, where more participants with IBS rated white flour as unhealthy compared to the non-IBS group (*p* = 0.044), Fig. 2.

### Avoidance of foods or food components due to their perceived healthiness

**Fig. 3:**
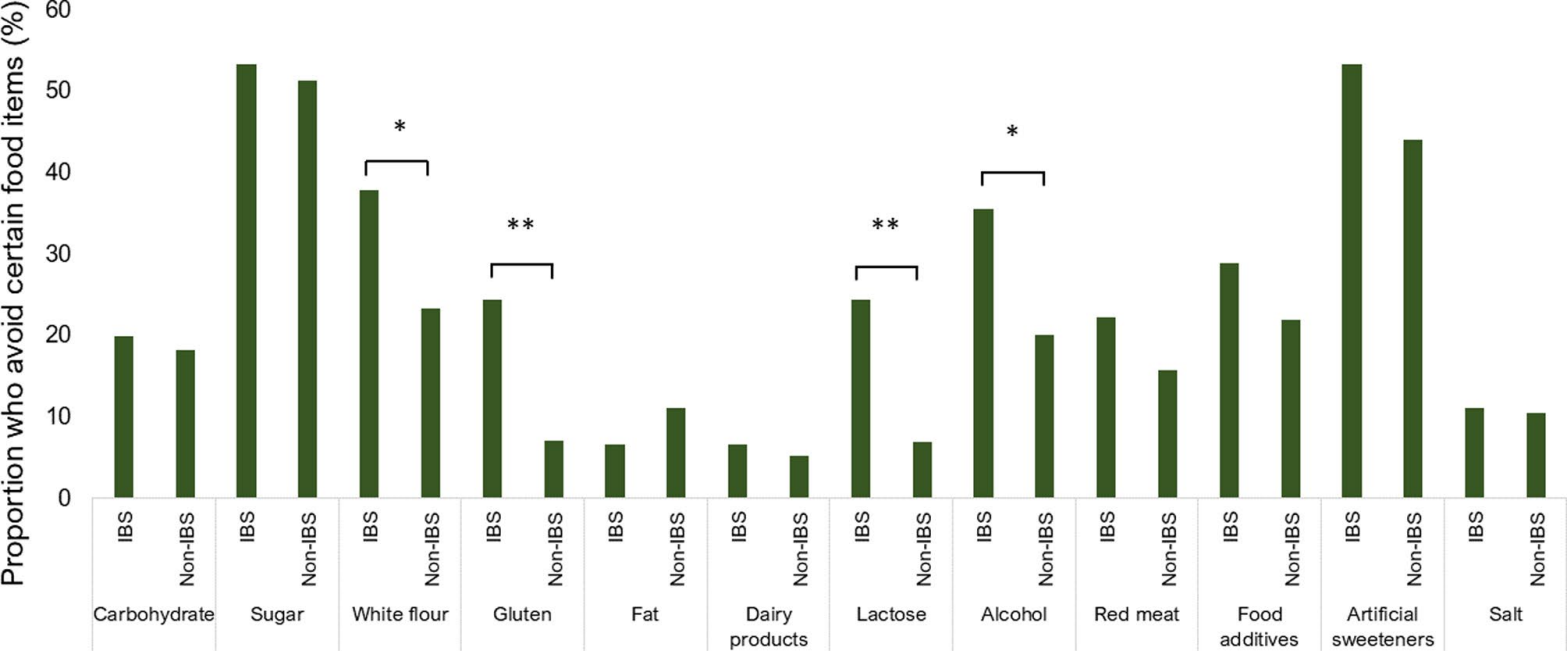
Reported avoidance of foods or food components because they were perceived to be unhealthy among participants with irritable bowel syndrome (IBS), *N* = 45, and participants without IBS, *N* = 493. *p values < 0.05 **p values < 0.001

Reported avoidance of food items or food components was common and more prevalent among participants with IBS, where 91% reported avoiding certain foods due to perceiving them as unhealthy vs. 78% in the non-IBS group (*p* = 0.025). The most commonly avoided food items in the group as whole were sugar (52%) and artificial sweeteners (45%) (Fig. 3). Food items that were avoided to a significantly larger extent among participants with IBS were gluten (24% vs. 7%, *p* < 0.001), lactose (24% vs. 7%, *p* < 0.001), white flour (38% vs. 23%, *p* = 0.028) and alcohol (36% vs. 20%, *p* = 0.016).

In univariable logistic regression analyses, after excluding those who reported being lactose or gluten intolerant, there was a > 6 times higher OR of excluding lactose (*p* < 0.001) and 3 times higher OR of excluding gluten (*p* = 0.007) among participants with IBS (Table 2). After adjusting for sex and BMI, the OR remained statistically significant for lactose, gluten, and alcohol. Furthermore, after adjusting for abdominal pain or discomfort, the models were no longer statistically significant.

**Table 2:** Differences in food avoidance due to perceived unhealthiness reported by participants with irritable bowel syndrome (IBS) *N* = 45 compared to non-IBS participants *N* = 493

	Non-IBS	IBS - Univariable		IBS – Model 1^a^		IBS – Model 2^b^	
	OR (ref)	OR (CI)	*p* value	OR (CI)	*p* value	OR (CI)	*p* value
Carbohydrate	1.0	1.11 (0.52–2.41)	0.77	1.02 (0.46–2.25)	0.96	0.94 (0.38–2.34)	0.94
Sugar	1.0	1.08 (0.59-2.00)	0.79	1.09 (0.58–2.03)	0.79	1.23 (0.60–2.50)	0.57
White flour	1.0	2.00 (1.06–3.78)	0.034	1.62 (0.84–3.11)	0.15	1.63 (0.76–3.51)	0.21
White flour^c^	1.0	1.76 (0.88–3.54)	0.11	1.46 (0.71–2.99)	0.30	1.58 (0.69–3.63)	0.28
Gluten	1.0	4.23 (1.98–9.07)	< 0.001	3.33 (1.53–7.28)	0.003	1.97 (0.77–5.06)	0.16
Gluten^c^	1.0	3.43 (1.40–8.44)	0.007	2.96 (1.17–7.45)	0.022	1.85 (0.61–5.57)	0.28
Fat	1.0	0.57 (0.17–1.90)	0.36	0.61 (0.18–2.07)	0.42	0.58 (0.15–2.24)	0.43
Dairy products	1.0	1.28 (0.37–4.42)	0.69	1.08 (0.31–3.82)	0.91	0.61 (0.15–2.51)	0.49
Lactose	1.0	4.37 (2.04–9.38)	< 0.001	4.45 (2.00-9.87)	< 0.001	1.95 (0.76-5.00)	0.17
Lactose^d^	1.0	6.17 (2.04–18.65)	< 0.001	6.36 (1.98–20.53)	0.002	3.78 (0.87–16.44)	0.076
Alcohol	1.0	2.20 (1.15–4.20)	0.018	2.02 (1.03–3.95)	0.041	1.31 (0.62–2.85)	0.50
Red meat	1.0	1.52 (0.72–3.20)	0.27	1.16 (0.54–2.49)	0.70	0.91 (3.76–2.19)	0.83
Food additives	1.0	1.45 (0.73–2.86)	0.29	1.13 (0.56–2.25)	0.74	1.35 (0.60–3.04)	0.48
Artificial sweeteners	1.0	1.45 (0.79–2.67)	0.24	1.33 (0.71–2.47)	0.38	1.39 (0.68–2.84)	0.37
Salt	1.0	1.06 (0.40–2.81)	0.91	1.06 (0.40–2.87)	0.90	0.74 (0.24–2.27)	0.60

*Abbreviations* OR, odds ratio; CI, confidence interval

^a^ Model 1 adjusted for sex, and body mass index

^b^ Model 2 adjusted for sex, body mass index, and abdominal pain

^c^ Individuals with reported gluten intolerance were excluded

^d^ Individuals with reported lactose intolerance were excluded

### Worry or anxiety related to diet healthiness

**Fig. 4:**
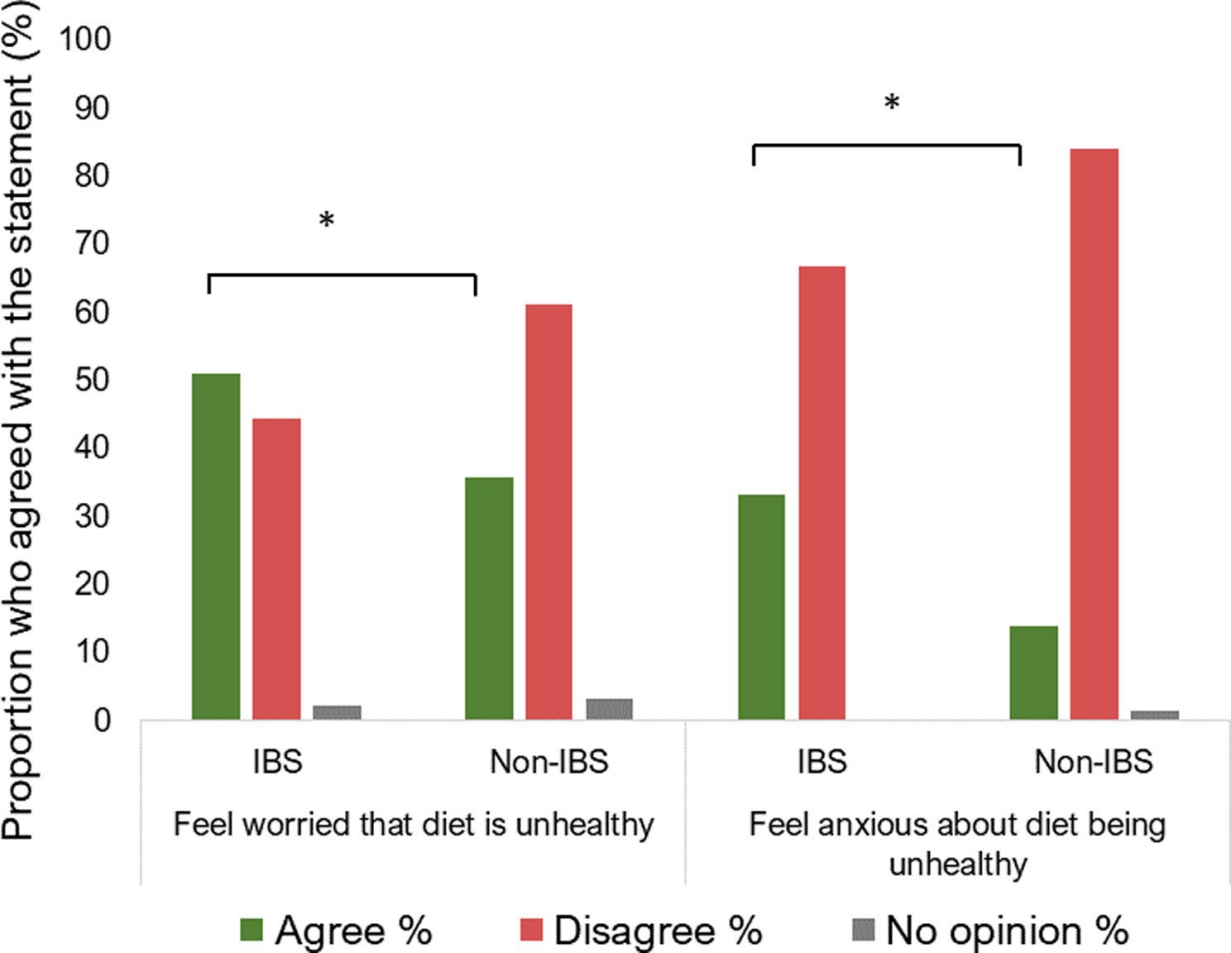
The proportion of participants with irritable bowel syndrome (IBS) who reported feeling worried or anxious about the healthiness of their diet was significantly larger than that of participants without IBS. Participants with IBS *N* = 45, and participants without IBS, *N* = 493. *p values < 0.05

More than half of the participants with IBS (51%) reported feeling worried about the healthiness of their diet, compared to 36% in the non-IBS group (*p* = 0.035) (Fig. 4). Furthermore, one-third (33%) of the participants with IBS reported feeling anxiety regarding the healthiness of their diet, which was significantly more prevalent compared to 14% in the non-IBS group (*p* = 0.002).

## Discussion

In this study, we found few differences in how individuals with IBS perceive the healthiness of foods compared to individuals without IBS. Food avoidance was more common in participants with IBS. Specifically, avoidance of lactose, gluten, and alcohol was more frequently reported in individuals with IBS than in those without IBS, but avoidance seemed to be driven primarily by abdominal pain. Worry and anxiety concerning the healthiness of diet were more prevalent among individuals with IBS than among those without IBS, with anxiety being especially pronounced.

Attitudes toward foods in regard to their perceived healthiness have not previously been described in patients with IBS. The results of this study show few differences between individuals with IBS and non-IBS individuals regarding the perceptions of the health aspects of food or food components, namely with white flour being the only exception. White flour is complex in the sense that it contains gluten, fructans (i.e., a FODMAP), and other wheat proteins, such as amylose trypsin inhibitors, and is low in dietary fiber [22]. Wheat products have been consistently reported to aggravate symptoms of IBS [23] and have frequently been reported as a food component that individuals with IBS avoid eating to manage their IBS symptoms [18, 24, 25]. In this study, we specifically asked if participants avoided foods because they perceived them as unhealthy and not for the sake of avoiding gastrointestinal symptoms, and nine out of ten individuals with IBS reported avoiding certain foods due to perceiving them as unhealthy. However, it remains unclear whether participants with IBS equated foods that trigger their gastrointestinal symptoms as being “unhealthy” in a broader sense. For example, a food that causes discomfort may be perceived as harmful to their overall health, even if it might conventionally be labeled as healthy for the general population. In individuals with IBS, the concept of healthiness may also be connected to the absence of adverse gastrointestinal reactions rather than solely the nutritional value.

Food avoidance is a known, but somewhat problematic, strategy among patients with IBS to manage their IBS symptoms [26]. In one study, 13.2% of patients reported severe food avoidance and restriction, and these patients had lower caloric intakes and lower intake of several micronutrients compared to patients without food avoidant behaviors [18]. In our study, participants with IBS reported avoiding gluten, lactose, white flour, and alcohol more frequently than participants without IBS. Notably, we observed a sixfold higher odds of lactose avoidance among participants with IBS, even after excluding individuals who reported being lactose intolerant. Lactose, a disaccharide and FODMAP component, is known to exacerbate symptoms in many individuals, particularly those with IBS [27]. The reason why individuals who do not perceive themselves as lactose intolerant still avoid lactose remains unclear, though a combination of psychological factors and adverse reactions to dairy products is likely involved [28]. However, we did not observe differences in dairy product avoidance between participants with and without IBS, which may be due to the increased availability of lactose-free dairy products in Sweden over the past decade.

Alcohol was also reported as avoided more frequently by the IBS group, and this corresponds well with previous research that has shown that alcohol or alcoholic beverages are commonly avoided by individuals with IBS [29]. In a study of Swedish adults with IBS, 31% of IBS patients avoided wine and beer [17]. However, after adjusting for abdominal pain, we no longer observed that IBS itself was a significant determinant of food avoidance. Abdominal pain is a key feature of IBS but was also commonly reported in our study population among individuals without IBS. Having abdominal pain, regardless of IBS, seems to be driving avoidance of certain foods.

In other studies of food avoidance in individuals with IBS, commonly reported avoided foods or food components include dairy products, legumes, apple, white flour, gluten-containing foods, spicy foods, and high-fat foods [17, 30]. Here, we did not note any significant avoidance of fat. This may reflect the spirit of the time when the survey was conducted, as high-fat diets, often abundant in dairy products, had been popular in Sweden for a period of time and thus might not have been perceived as unhealthy.

Anxiety and depression are known to be more prevalent in IBS patients than in the general population [11]. The increased risk of anxiety or depression is, however, also increased among individuals with functional abdominal pain without fulfilling requirements for IBS diagnosis [31]. Anxiety in general, as well as GI-specific anxiety (worry and/or awareness of abdominal discomfort), has been associated with more severe IBS symptoms [17]. In this study, we also showed that patients with IBS felt more worried and anxious in regards to the healthiness of their diet compared to non-IBS individuals. This might pose an additional burden on the patients and could potentially lead to even more severe food avoidance. However, due to the small number of participants, we could not assess whether individuals who felt worried about the healthiness of their diet had an increased risk of food avoidance.

### Strengths and limitations

The methodological strengths of this study include the random selection of participants, a fairly high response rate for a postal survey [32, 33], and a high completion rate among respondents. However, as with all dietary studies, there may have been a selection bias, as individuals with a greater interest in health and diet may have been more likely to respond, which may limit the generalizability of the results. In addition, there was a slight overrepresentation of women among the respondents and a somewhat higher proportion of individuals with higher education compared to the general Swedish population, which should be considered when interpreting the results. The study only included individuals aged 20–65 years. However, that does not reduce external validity, since IBS is less common outside of this age range [34].

A limitation in this study is the somewhat small sample size of participants included with IBS, reducing the power to detect true differences between the groups and preventing further sensitivity analyses. A more balanced sample size between participants with and without IBS would have been optimal. Additionally, the diagnosis of IBS was self-reported and, due to the nature of the survey, could not be confirmed by a physician [35]. However, the proportion of individuals reporting IBS closely matched the expected prevalence rate, which enhances the credibility of these reports [3]. Furthermore, we strengthened the validity of the self-reported diagnosis by including a control question about the presence of frequent abdominal pain and/or discomfort, further supporting the accuracy of the IBS diagnosis. Optimally we would have included all diagnostic criteria for IBS in the questionnaire, but since we had to restrict the number of questions included, this was not possible. Moreover, all foods and food components assessed in this study were predefined, which may have left out foods of importance.

## Conclusion

This study found that individuals with IBS are more likely to avoid gluten, lactose, and alcohol compared to those without IBS. Notably, abdominal pain seems to drive avoidance of foods, regardless of having IBS or not. Additionally, worry and anxiety related to diet are more common in individuals with IBS. Given the lack of validated tools to measure food-related anxiety, we emphasize the need for the development of such tools to better investigate the underlying causes of food-related anxiety in IBS. Understanding the interactions between symptom burden, dietary habits, and psychological distress is essential for optimizing treatment strategies for this patient group.

## Electronic supplementary material

Below is the link to the electronic supplementary material.

Supplementary Material 1

## Data Availability

The data that support the findings of this study are not openly available as they are subject to secrecy in accordance with the Swedish Public Access to Information and Secrecy Act (Offentlighets- och sekretesslagen, OSL, 2009:400) but can be made available to researchers upon request (subject to a review of secrecy). Requests for data should be made to Linnea Bärebring (linnea.barebring@gu.se). Data are located in controlled access data storage at the University of Gothenburg.
